# Supplementary material for: Prevalence and associated factors of overweight and obesity among Afghan school children: A cross-sectional analytical study from Kandahar City, Afghanistan
Source: PLoS One. 2025 Mar 17;20(3):e0320039. doi: 10.1371/journal.pone.0320039 (PMC11913295; doi:10.1371/journal.pone.0320039)
Supplement: Attachment — Submitted filename: PONE-D-24-38181.pdf [file pone.0320039.s001.pdf]

PLOS ONE

Prevalence and associated factors of overweight and obesity among Afghan school children: a cross-sectional analytical study from Kandahar city, Afghanistan.

--Manuscript Draft--

**Manuscript Number:**	PONE-D-24-38181
**Article Type:**	Research Article
**Full Title:**	Prevalence and associated factors of overweight and obesity among Afghan school children: a cross-sectional analytical study from Kandahar city, Afghanistan.
**Short Title:**	Overweight and obesity among Afghan school children.

**Corresponding Author:**	Bilal Ahmad Rahimi, M.D., D.T.M.&H., M.C.T.P., Ph.D<br>Kandahar University Faculty of Medicine<br>Kandahar, AFGHANISTAN

**Keywords:**	Obesity; overweight; Afghanistan; Kandahar; Risk factors; School children; Prevalence

Abstract:

Background

Childhood overweight and obesity is an emerging public health problem in developing countries. This is the first school-based study of its type from Afghanistan to estimate the prevalence and associated factors of overweight and obesity among Afghan school children aged 6–18 years in Kandahar city of Afghanistan.

Methods

This cross-sectional analytical study was conducted among 2281 school children from January–April, 2024. Sociodemographic, anthropometric measurements, and other data were collected from all the participants. Data were analyzed by using descriptive statistics, Chi square test, and multivariate logistic regression analysis.

Results

Among the 2281 enrolled children, 72.5% were boys, 65.1% going to private schools, and 53.8% poor. Prevalence of overweight and obesity was 11.5% (6.6% were overweight and 4.9% were obese). Mean age was 12.7 years. By logistic regression analysis, statistically significant risk factors associated w[illegible]verweight and obesity were being boy (AOR 1.5 and 95% CI 1.1–2.0), (ii) student of private school (AOR 2.2 and 95% CI 1.5–2.8), (iii) belonging to rich family (AOR 1.9 and 95% CI 1.3–2.7), (iv) parental obesity (AOR 1.5 and 95% CI 1.1–2.0).

Conclusion

School children of Kandahar city are suffering from overweight/obesity. In Afghanistan, the currently focused issue is only undernutrition. However, based on the results of this study, overweight/obesity should be dealt with as an emerging problem in school children of Kandahar city. It is recommended that Afghanistan ministries of education and public health, with the help of international donor agencies, such as WHO and UNICEF, work together in controlling overweight and obesity alongside the currently-focused undernutrition in school children of Kandahar city. Periodic special awareness programs on the prevention and control of overweight/obesity should be conducted in schools, radio, television, and other sources of social media.

Order of Authors:

Bilal Ahmad Rahimi, M.D., D.T.M.&H., M.C.T.P., Ph.D

Aziz Ahmad Khalid

Wahid Ahmad Khalid

Javed Ahmad Rahimi

Walter R Taylor

Powered by Editorial Manager® and ProduXion Manager® from Aries Systems Corporation

Additional Information:	
**Question**	**Response**
**Financial Disclosure**<br><br>Enter a financial disclosure statement that describes the sources of funding for the work included in this submission. Review the submission guidelines for detailed requirements. View published research articles from *PLOS ONE* for specific examples.<br><br>This statement is required for submission and **will appear in the published article** if the submission is accepted. Please make sure it is accurate.<br><br>**Funded studies**<br>Enter a statement with the following details:<br>• Initials of the authors who received each award<br>• Grant numbers awarded to each author<br>• The full name of each funder<br>• URL of each funder website<br>• Did the sponsors or funders play any role in the study design, data collection and analysis, decision to publish, or preparation of the manuscript?<br><br>Did you receive funding for this work?	The author(s) received no specific funding for this work.
**Competing Interests**<br><br>Use the instructions below to enter a competing interest statement for this submission. On behalf of all authors, disclose any competing interests that could be perceived to bias this work—acknowledging all financial support and any other relevant financial or non-financial competing interests.<br><br>This statement is required for submission and **will appear in the published article** if the submission is accepted. Please make sure it is accurate and that any funding sources listed in your Funding Information	All the authors do not have any competing interests.

Powered by Editorial Manager® and ProduXion Manager® from Aries Systems Corporation

later in the submission form are also declared in your Financial Disclosure statement.<br><br>View published research articles from *PLOS ONE* for specific examples.<br><br>**NO authors have competing interests**<br><br>Enter: *The authors have declared that no competing interests exist.*<br><br>**Authors with competing interests**<br><br>Enter competing interest details beginning with this statement:<br><br>*I have read the journal's policy and the authors of this manuscript have the following competing interests: [insert competing interests here]*<br><br>* typeset	
**Ethics Statement**<br><br>Enter an ethics statement for this submission. This statement is required if the study involved:<br><br>• Human participants<br>• Human specimens or tissue<br>• Vertebrate animals or cephalopods<br>• Vertebrate embryos or tissues<br>• Field research<br><br>Write "N/A" if the submission does not require an ethics statement.<br><br>General guidance is provided below. Consult the submission guidelines for detailed instructions. **Make sure that all information entered here is included in the Methods section of the manuscript.**	Consent to participate<br>Prior to the data collection, written informed consent from parents, and written informed assent from study participants were obtained with the help of school authorities. Interviews of participants using a predesigned questionnaire were conducted and anthropometric measurements were done as per standard operating procedures. For data collection, only patients' initials were used. Information of the participants would not be disclosed. Prior to entering into the computer for analysis, the collected data was coded and de-identified.<br><br>Ethical Approval<br>Ethical clearance was obtained from Ethics Committee of Kandahar University (code number KDRU-EC-2023.17) and permission from Kandahar Province Education Department authorities.

Powered by Editorial Manager® and ProduXion Manager® from Aries Systems Corporation

**Format for specific study types**<br><br>**Human Subject Research (involving human participants and/or tissue)**<br>• Give the name of the institutional review board or ethics committee that approved the study<br>• Include the approval number and/or a statement indicating approval of this research<br>• Indicate the form of consent obtained (written/oral) or the reason that consent was not obtained (e.g. the data were analyzed anonymously)<br><br>**Animal Research (involving vertebrate animals, embryos or tissues)**<br>• Provide the name of the Institutional Animal Care and Use Committee (IACUC) or other relevant ethics board that reviewed the study protocol, and indicate whether they approved this research or granted a formal waiver of ethical approval<br>• Include an approval number if one was obtained<br>• If the study involved *non-human primates*, add *additional details* about animal welfare and steps taken to ameliorate suffering<br>• If anesthesia, euthanasia, or any kind of animal sacrifice is part of the study, include briefly which substances and/or methods were applied<br><br>**Field Research**<br><br>Include the following details if this study involves the collection of plant, animal, or other materials from a natural setting:<br>• Field permit number<br>• Name of the institution or relevant body that granted permission	
**Data Availability**<br><br>Authors are required to make all data underlying the findings described fully available, without restriction, and from the time of publication. PLOS allows rare exceptions to address legal and ethical concerns. See the PLOS Data Policy and FAQ for detailed information.	Yes - all data are fully available without restriction

A Data Availability Statement describing where the data can be found is required at submission. Your answers to this question constitute the Data Availability Statement and **will be published in the article**, if accepted.<br><br>**Important:** Stating 'data available on request from the author' is not sufficient. If your data are only available upon request, select 'No' for the first question and explain your exceptional situation in the text box.<br><br>Do the authors confirm that all data underlying the findings described in their manuscript are fully available without restriction?	
**Describe where the data may be found in full sentences. If you are copying our sample text, replace any instances of XXX with the appropriate details.**<br><br>• If the data are **held or will be held in a public repository**, include URLs, accession numbers or DOIs. If this information will only be available after acceptance, indicate this by ticking the box below. For example: *All XXX files are available from the XXX database (accession number(s) XXX, XXX.).*<br>• If the data are all contained **within the manuscript and/or Supporting Information files**, enter the following: *All relevant data are within the manuscript and its Supporting Information files.*<br>• If neither of these applies but you are able to provide **details of access elsewhere**, with or without limitations, please do so. For example:<br><br>*Data cannot be shared publicly because of [XXX]. Data are available from the XXX Institutional Data Access / Ethics Committee (contact via XXX) for researchers who meet the criteria for access to confidential data.*<br><br>*The data underlying the results presented in the study are available from (include the name of the third party*	All relevant data are within the manuscript.

and contact information or URL).<br>• This text is appropriate if the data are owned by a third party and authors do not have permission to share the data.<br><br>* typeset	
Additional data availability information:	

Powered by Editorial Manager® and ProduXion Manager® from Aries Systems Corporation

Manuscript file
Click here to access/download;Manuscript;Manuscript (overweight and obesity among school children).docx

Title Page

Title of the Article: Prevalence and associated factors of overweight and obesity among Afghan school children: a cross-sectional analytical study from Kandahar city, Afghanistan.

Running title: Overweight and obesity among Afghan school children.

Authors

1. Bilal Ahmad Rahimi

   MD, DTM&H, MCTM, PhD

   Professor, Department of Pediatrics, Faculty of Medicine, Kandahar University, Kandahar, Afghanistan.

   Email: drbilal77@yahoo.com

   ORCID**:** 0000-0002-6630-5742

2. Aziz Ahmad Khalid

   Department of Economics, Jamia Millia Islamia, Central University, New Delhi, India. azizkhalid24@gmail.com

3. Wahid Ahmad Khalid

   Department of Economics, Savitribai Phule Pune University, Pune, Maharashtra, India. wahid.khalid@outlook.com

4. Javed Ahmad Rahimi

   Department of Business Administration, Gujarat University, Ahmedabad, Gujarat, India. Javedahmadrahimi560@gmail.com

5. Walter R Taylor

   MD, FRCP

   Associate Professor, Mahidol Oxford Tropical Medicine Clinical Research unit (MORU), Mahidol University, Bangkok, Thailand

Email: Bob@tropmedres.ac

Corresponding author:

Bilal Ahmad Rahimi

MD, DTM&H, MCTM, PhD

E-mail address: drbilal77@yahoo.com

Telephone number: +93700309692

ORCID: 0000-0002-6630-5742

Designation: Prof. and Head, Department of Pediatrics, Faculty of Medicine, Kandahar University, Kandahar, Afghanistan.

Faculty of Medicine, Afghan International Islamic University, Kabul, Afghanistan.

Postal address: Department of Pediatrics, Faculty of Medicine, Kandahar University, Durahi, Beside Aino Mena Town, District 10, Kandahar, Afghanistan.

Abstract

Background

Childhood overweight and obesity is an emerging public health problem in developing countries. This is the first school-based study of its type from Afghanistan to estimate the prevalence and associated factors of overweight and obesity among Afghan school children aged 6–18 years in Kandahar city of Afghanistan.

Methods

This cross-sectional analytical study was conducted among 2281 school children from January 10–April 15, 2024. Sociodemographic, anthropometric measurements, and other data were collected from all the participants. Data were analyzed by using descriptive statistics, Chi square test, and multivariate logistic regression analysis.

Results

Among the 2281 enrolled children, 72.5% were boys, 65.1% going to private schools, and 53.8% poor. Prevalence of overweight and obesity was 11.5% (6.6% were overweight and 4.9% were obese). Mean age was 12.7 years. By logistic regression analysis, statistically significant risk factors associated with overweight and obesity were being boy (AOR 1.5 and 95% CI 1.1–2.0), (ii) student of private school (AOR 2.2 and 95% CI 1.5–2.8), (iii) belonging to rich family (AOR 1.9 and 95% CI 1.3–2.7), (iv) parental obesity (AOR 1.5 and 95% CI 1.1–2.0).

Conclusion

School children of Kandahar city are suffering from overweight/obesity. In Afghanistan, the currently focused issue is only undernutrition. However, based on the results of this study, overweight/obesity should be dealt with as an emerging problem in school children of Kandahar city. It is recommended that Afghanistan ministries of education and public health, with the help of international donor agencies, such as WHO and UNICEF, work together in controlling overweight and obesity alongside the currently-focused undernutrition in school children of Kandahar city. Periodic special awareness programs on the prevention and control of overweight/obesity should be conducted in schools, radio, television, and other sources of social media.

Key words

Obesity; Overweight; Afghanistan; Kandahar; Risk factors; School children; Prevalence.

Introduction

Globally, there has been a significant increase in the prevalence of overweight and obesity among primary school children in both developed and developing countries [1–3]. In 2016, worldwide 124 million children aged 5–19 years were estimated to be suffering from obesity [4]. A study conducted in USA revealed that 31.0% of the children were overweight [5]. A study conducted in Italy showed that the prevalence of overweight/obesity among children was 25.2% [6]. In India, the prevalence of overweight among boys and girls was 18% and 16%, respectively [7].

Childhood overweight and obesity is an emerging public health problem in developing countries [8]. This is largely related with the increasing westernization of societies and associated changes in lifestyle [9]. Many developing countries are currently in a state of nutritional transition, i.e., there is a persistently increased prevalence of under-nutrition in the face of emerging overweight and obesity [10–11]. Overweight/obesity causes complications during childhood and adolescence which persist into adulthood, increasing the risk of morbidity and mortality later in life [12]. These complications are the development of high blood pressure, long-term cardiovascular morbidities, and early death [8].

Some studies have reported that environmental and genetic factors affect the prevalence of overweight and obesity, although some inconsistencies in the effects have been reported [13–14]. Major risk factors of overweight/obesity among children, especially in developing countries, are belonging to rich family, higher levels of maternal education, decreased physical activity, female gender, and race [15–16]. In childhood, overweight and obesity are amenable to treatment. So, early intervention could help in reducing both short- and long-term complications of overweight and obesity, as well as break the tracking into adolescence and adulthood [17].

Very few studies among children have been reported from Kandahar province of Afghanistan [18–23]. To our knowledge, regarding overweight and obesity among adolescents, only two studies have been reported from the entire Afghanistan. In 2014, global school-based student health survey (GSHS) reported that the prevalence of overweight was 19.0% among 1493 students aged 12–15 years [24]. However, Afghanistan national nutrition survey 2013 revealed that 10.4% of 6392 adolescent girls aged 10–19 years were overweight or obese in Afghanistan [25].

To the level of our knowledge, there is not publish study from the entire Afghanistan that study the prevalence and associated factors of overweight and obesity among school children aged 6–18 years. So, this is the first school-based study of its type not only from Kandahar city but the whole Afghanistan to estimate the prevalence and associated factors of overweight and obesity among Afghan school children in Kandahar city of Afghanistan.

Materials and Methods

Study design and study area

This was a school-based cross-sectional analytical study, conducted during four-month-period (January 10–April 15, 2024). Kandahar is the second largest city, located in the south-west of Afghanistan. This city has an altitude of 1010 meters above sea level and a population of approximately 614118 people. Among the school of Kandahar city, ten schools (five government and five private schools) were randomly selected for this study using lottery method of randomization.

Study population and sample size calculation

The study population of this research was composed of school children aged 6–18 years and permanent residents of Kandahar. All those children were excluded from the study whose parent/guardian refused to participate in the study. The demographic characteristics of the excluded children were similar to the included children. Also, during the selection process, the socioeconomic, dietary, distance between home to school, and other factors that may affect the overweight/obesity rate were taken in consideration. So, there was no representative bias in this study.

Sample size was calculated using software of Epi Info version 7.2.2.6 (CDC, Atlanta, Georgia, USA). A non-response rate of 20% was added. So, our sample size was 2395 school children. Among these children, 114 (4.8%) were excluded because their parents/guardians refused to take part in the study. The response rate was 95.2%. So, we collected data from 2281 school children.

Sample collection, data quality management, and anthropometric measurements in children

For this study, children of the ten schools were selected using lottery method of randomization. A researcher-made questionnaire was used in two local languages (Pashto and Dari) with questions regarding socio-demographic characteristics, anthropometric measurements, and other characteristics for the study. Paper-based data collection was conducted by experienced and trained researchers. In each school, before starting the data collection, all children were provided short information about the objectives and methods of this study. All the data collectors were well trained on how to conduct the interview with school children and their parents/guardians and how to do the anthropometric measurements.

All methods were performed in accordance with the relevant guidelines and regulations. Data of anthropometric measurements such as body weight and height were collected from each child. All children stood barefooted against a vertical wall and height was obtained using a stadiometer to the nearest 0.1 cm. Body weight of children with lightweight clothes was measured to the nearest 0.1 kg using a digital balance which was validated before starting the measurement of weight. About 10% of the measurements were randomly selected for quality control and measured by another experienced researcher who was blinded for the previous measurement results. Besides, data was double entered to minimize the errors.

In this study, poverty was defined as family which earns <155 Afghanis (<2.15 USD) per person per day [26]. For nutritional status, anthropometric indices such as height-for-age Z (HAZ) score, weight-for-age Z (WAZ) score, weight-for-height Z (WHZ) score, and BMI-for-age Z (BAZ) score were computed as per the 2009 WHO Child Growth Standards median to assess the growth and nutritional status of the children [27]. Children were defined as normal if their WAZ score was –2 SD to +2 SD or HAZ score –2 SD to +2 SD or BAZ score –2 SD to +1 SD of WHO Child Growth Standards median. Children were defined as undernourished if their WAZ score was < –2 SD (underweight) or HAZ score < –2 SD (stunting) or BAZ score < –2 SD (thinness) of WHO Child Growth Standards median. Children were defined as overweight if their BAZ score was > +1 SD of WHO Child Growth Standards median.

Children were defined as obese if their BAZ score was > +2 SD of WHO Child Growth Standards median [27].

Ethical considerations

Ethical approval was obtained from Ethics Committee of Kandahar University (code number KDRU-EC-2023.17) and permission from Kandahar Province Education Department authorities. Prior to the data collection, written informed consent from parents, and written informed assent from study participants were obtained. Interviews of the study participants using a predesigned questionnaire were conducted. For data collection, only children initials were used. Information of the participants would not be disclosed. Prior to entering into the computer for analysis, the collected data were coded and de-identified.

Data analysis

The data were entered into Microsoft Excel 2021, cleaned, and imported to Statistical Package for the Social Sciences (SPSS) version 25 (Chicago, IL, USA) for statistical analysis. Independent variables in this study included sociodemographic factors, physical activity, and food consumption patterns of school children. Descriptive analysis including frequency, percentage, mean, standard deviation (SD), and range was used to summarize independent variables and prevalence of overweight/obesity among school children. For bivariate analysis, Chi-square test (using crude odds ratio [COR]) was performed to determine association between the independent and dependent variables. All the variables that were statistically significant in bivariate analysis were adjusted and assessed for independence in a multivariate logistic regression (using adjusted odds ratio [AOR]) to determine the associated factors of overweight/obesity among school children. A p-value of <0.05 was considered statistically significant.

Results

This study was conducted during January–April 2024 in 2281 Afghan school children with mean (SD) age of 12.7 (2.1) years. In these children, 72.5% (1654/2281) were boys, 65.1% (1484/2281) in private schools, and 53.8% (1227/2281) having a family monthly income of <10,000 Afghanis. Parental obesity

was present in 28.6% (652/2281) children while 67.2% (1739/2281) children going to and from school using motorcycle, car, or bus. Among these children, the prevalence of obesity, overweight, underweight, and normal BMI were present in 4.9% (112/2281), 6.6% (150/2281), 51.7% (1179/2281), and 36.8% (840/2281), respectively (Table 1). Comparison of main sociodemographic and other characteristics of the school children between government vs private schools are described in Table 2.

Table 1. Sociodemographic, physical activities, and food consumption patterns of the study participants with comparison between boys and girls.

Variable	Frequency (%), N=2281	Boys N=1654	Girls N=627	*P*-value
**Age**				
6–11 years	882 (38.7)	636 (72.1)	246 (27.9)	0.732
12–18 years	1399 (61.3)	1018 (72.8)	381 (27.2)	
**School type**				
Government	797 (34.9)	636 (79.8)	161 (20.2)	<0.001
Private	1484 (65.1)	1018 (68.6)	466 (31.4)	
**Residence ownership**				
Owned	1542 (67.6)	1061 (68.8)	481 (31.2)	<0.001
Rented	739 (32.4)	593 (80.2)	146 (19.8)	
**Family economic status**				
Not rich	2065 (90.5)	1489 (72.1)	576 (27.9)	0.180
Rich	216 (9.5)	165 (76.4)	51 (23.6)	
**Father's literacy**				
Literate	471 (20.6)	368 (78.1)	103 (21.9)	0.002
Illiterate	1810 (79.4)	1286 (71.0)	524 (29.0)	
**Mother's literacy**				
Literate	420 (18.4)	274 (65.2)	146 (34.8)	<0.001
Illiterate	1861 (81.6)	1380 (74.2)	481 (25.8)	
**Father occupation**				
Employed	1989 (87.2)	1459 (73.4)	530 (26.6)	0.019
Unemployed	292 (12.8)	195 (66.8)	97 (33.2)	
**Mother occupation**				
Employed	78 (3.4)	58 (74.4)	20 (25.6)	0.710
Unemployed/Housewife	2203 (96.6)	1596 (72.4)	607 (27.6)	
**Parental obesity**				
Yes	652 (28.6)	505 (77.5)	147 (22.5)	0.001
No	1629 (71.4)	1149 (70.5)	480 (29.5)	
**Parental hypertension**				
Yes	198 (8.7)	162 (81.8)	36 (18.2)	0.002
No	2083 (91.3)	1492 (71.6)	591 (28.4)	
**Parental diabetes mellitus**				
Yes	70 (3.1)	48 (68.6)	22 (31.4)	0.453

No	2211 (96.9)	1606 (72.6)	605 (27.4)	
**Modes of transport to and from school**<br>Walking/Bicycle<br>Motorcycle/car/bus	 542 (23.8)<br>1739 (76.2)	 386 (71.2)<br>1268 (72.9)	 156 (28.8)<br>471 (27.1)	0.440
**Doing exercise/playing outdoor game**<br>Always/mostly<br>Sometimes/rarely	 724 (31.7)<br>1557 (68.3)	 521 (72.0)<br>1133 (72.8)	 203 (28.0)<br>424 (27.2)	0.688
**Time spent daily on watching television**<br>< 2 hours<br>≥ 2 hours	 419 (18.4)<br>1862 (81.6)	 302 (72.1)<br>1352 (72.6)	 117 (27.9)<br>510 (27.4)	0.825
**Consumption of snacks in between meals**<br>Always/mostly<br>Sometimes/rarely	 1318 (57.8)<br>963 (42.2)	 949 (72.0)<br>705 (73.2)	 369 (28.0)<br>258 (26.8)	0.524
**Eating confectionery/sweet foods**<br>Always/mostly<br>Sometimes/rarely	 575 (25.2)<br>1706 (74.8)	 412 (71.7)<br>1242 (72.8)	 163 (28.3)<br>464 (27.2)	0.593
**Drinking soft drinks**<br>Always/mostly<br>Sometimes/rarely	 1481 (64.9)<br>800 (35.1)	 1085 (73.3)<br>569 (71.1)	 396 (26.7)<br>231 (28.9)	0.275
**Frequency of consuming vegetables**<br>Always/mostly<br>Sometimes/rarely	 1273 (55.8)<br>1008 (44.2)	 921 (72.3)<br>733 (72.7)	 352 (27.7)<br>275 (27.3)	0.844
**Frequency of consuming fruit**<br>Always/mostly<br>Sometimes/rarely	 362 (15.9)<br>1919 (84.1)	 292 (80.7)<br>1362 (71.0)	 70 (19.3)<br>557 (29.0)	<0.001
**Overweight/obesity**<br>Yes<br>No	 262 (11.5)<br>2019 (88.5)	 205 (78.2)<br>1449 (71.8)	 57 (21.8)<br>570 (28.2)	0.027

* Family which earn <155 Afghanis (<2.15 USD) per person per day.

Table 2. Main sociodemographic and other characteristics of the study participants with comparison between government and private school students.

Variable	Frequency n (%) N=2281	Government school n (%) N=797	Private school n (%) N=1484	*P*-value
**Age**<br>6–11 years<br>12–18 years	 882 (38.7)<br>1399 (61.3)	 332 (37.6)<br>465 (33.2)	 550 (62.4)<br>934 (66.8)	0.032

**Sex**				
Boys	1654 (72.5)	636 (38.5)	1018 (61.5)	<0.001
Girls	627 (27.5)	161 (25.7)	466 (74.3)	
**Residence ownership**				
Owned	1542 (67.6)	574 (37.2)	968 (62.8)	0.001
Rented	739 (32.4)	223 (30.2)	516 (69.8)	
**Family economic status**				
Not rich	2065 (90.5)	739 (35.8)	1326 (64.2)	0.009
Rich	216 (9.5)	58 (26.9)	158 (73.1)	
**Father's literacy**				
Literate	471 (20.6)	158 (33.5)	313 (66.5)	0.476
Illiterate	1810 (79.4)	639 (35.3)	1171 (64.7)	
**Mother's literacy**				
Literate	420 (18.4)	163 (38.8)	257 (61.2)	0.066
Illiterate	1861 (81.6)	634 (34.1)	1227 (65.9)	
**Father occupation**				
Employed	1989 (87.2)	690 (34.7)	1299 (65.3)	0.513
Unemployed	292 (12.8)	107 (36.6)	185 (63.4)	
**Mother occupation**				
Employed	78 (3.4)	34 (43.6)	44 (56.4)	0.103
Unemployed/Housewife	2203 (96.6)	763 (34.6)	1440 (65.4)	
**Parental obesity**				
Yes	652 (28.6)	266 (40.8)	386 (59.2)	<0.001
No	1629 (71.4)	531 (32.6)	1098 (67.4)	
**Parental hypertension**				
Yes	198 (8.7)	68 (34.3)	130 (65.7)	0.854
No	2083 (91.3)	729 (35.0)	1354 (65.0)	
**Parental diabetes mellitus**				
Yes	70 (3.1)	26 (37.1)	44 (62.9)	0.695
No	2211 (96.9)	771 (34.9)	1440 (65.1)	
**Modes of transport to and from school**				<0.001
Walking/Bicycle	542 (23.8)	259 (47.8)	283 (52.2)	
Motorcycle/car/bus	1739 (76.2)	538 (30.9)	1201 (69.1)	
**Doing exercise/playing outdoor game**				0.704
Always/mostly	724 (31.7)	257 (35.5)	467 (64.5)	
Sometimes/rarely	1557 (68.3)	540 (34.7)	1017 (65.3)	
**Time spent daily on watching television**				<0.001
< 2 hours	419 (18.4)	212 (50.6)	207 (49.4)	
≥ 2 hours	1862 (81.6)	585 (31.4)	1277 (68.6)	
**Consumption of snacks in between meals**				0.504
Always/mostly	1318 (57.8)	453 (34.4)	865 (65.6)	
Sometimes/rarely	963 (42.2)	344 (35.7)	619 (64.3)	
**Eating confectionery/sweet foods**				
Always/mostly	575 (25.2)	209 (36.3)	366 (63.7)	0.413
Sometimes/rarely	1706 (74.8)	588 (34.5)	1118 (65.5)	

**Drinking soft drinks**				
Always/mostly	1481 (64.9)	520 (35.1)	961 (64.9)	0.816
Sometimes/rarely	800 (35.1)	277 (34.6)	523 (65.4)	
**Frequency of consuming vegetables**				
Always/mostly	1273 (55.8)	457 (35.9)	816 (64.1)	0.281
Sometimes/rarely	1008 (44.2)	340 (33.7)	668 (66.3)	
**Frequency of consuming fruit**				
Always/mostly	362 (15.9)	149 (41.2)	213 (58.8)	0.007
Sometimes/rarely	1919 (84.1)	648 (33.8)	1271 (66.2)	
**Overweight/obesity**				
Yes	262 (11.5)	57 (21.8)	205 (78.2)	<0.001
No	2019 (88.5)	740 (36.7)	1279 (63.3)	

Based on Chi-square test analysis, statistically significant factors associated with overweight/obesity among school children were (i) being boy (COR 1.4, 95% CI 1.0–1.9, and *p*-value 0.027), (ii) student of private school (COR 2.1, 95% CI 1.5–2.8, and *p*-value <0.001), (iii) belonging to rich family (COR 2.1, 95% CI 1.5–3.0, and *p*-value <0.001), (iv) parental obesity (COR 1.4, 95% CI 1.1–1.9, and *p*-value 0.013), (v) using motorcycle/car/bus for transport to and from school (COR 1.8, 95% CI 1.3–2.6, and *p*-value 0.001), and (vi) spending ≥2 hours daily on watching television (COR 1.7, 95% CI 1.2–2.5, and *p*-value 0.006). Finally, based on logistic regression analysis, the independent statistically significant risk factors associated with overweight/obesity among school children were (i) being boy (AOR 1.5, 95% CI 1.1–2.0, and *p*-value 0.021), (ii) student of private school (AOR 2.2, 95% CI 1.5–2.8, and *p*-value <0.001), (iii) belonging to rich family (AOR 1.9, 95% CI 1.3–2.7, and *p*-value 0.001), (iv) parental obesity (AOR 1.5, 95% CI 1.1–2.0, and *p*-value 0.006) (Table 3).

Table 3. Chi-square and logistic regression analysis of the factors associated with overweight/obesity among the study participants.

Variable	Total n (%), N=2281	Overweight/obesity Present n (%), N=262	Overweight/obesity Absent n (%), N=2019	COR (95% CI)	*P*-value	AOR (95% CI)*	*P*-value
**Age**							
6–11 years	882 (38.7)	105 (11.9)	777 (88.1)	0.9 (0.7–1.2)	0.619	–	–
12–18 years	1399 (61.3)	157 (11.2)	1242 (88.8)	1			
**Sex**							
Boys	1654 (72.5)	205 (12.4)	1449 (87.6)	1.4 (1.0–1.9)	0.027	1.5 (1.1–2.0)	0.021
Girls	627 (27.5)	57 (9.1)	570 (90.9)	1		1	
**School type**							
Government	797 (34.9)	57 (7.2)	740 (92.8)	1	<0.001	1	<0.001
Private	1484 (65.1)	205 (13.8)	1279 (86.2)	2.1 (1.5–2.8)		2.2 (1.5–2.8)	
**Residence ownership**							
Owned	1542 (67.6)	175 (11.3)	1367 (88.7)	1	0.766	–	–
Rented	739 (32.4)	87 (11.8)	652 (88.2)	1.0 (0.8–1.4)			
**Family economic status**							
Not rich	2065 (90.5)	219 (10.6)	1846 (89.4)	1	<0.001	1	0.001
Rich	216 (9.5)	43 (19.9)	173 (80.1)	2.1 (1.5–3.0)		1.9 (1.3–2.7)	
**Father's literacy**							
Literate	471 (20.6)	37 (7.9)	434 (92.1)	1	0.006	–	–
Illiterate	1810 (79.4)	225 (12.4)	1585 (87.6)	0.6 (0.4–0.9)			
**Mother's literacy**							
Literate	420 (18.4)	42 (10.0)	378 (90.0)	1	0.290	–	–
Illiterate	1861 (81.6)	220 (11.8)	1641 (88.2)	1.2 (0.9–1.7)			
**Father occupation**							
Employed	1989 (87.2)	230 (11.6)	1759 (88.4)	1.1 (0.7–1.6)	0.762	–	–
Unemployed	292 (12.8)	32 (11.0)	260 (89.0)	1			
**Mother occupation**							
Employed	78 (3.4)	6 (7.7)	72 (92.3)	1	0.285	–	–
Unemployed/Housewife	2203 (96.6)	256 (11.6)	1947 (88.4)	1.6 (0.7–3.7)			

**Parental obesity**<br>Yes<br>No	652 (28.6)<br>1629 (71.4)	92 (14.1)<br>170 (10.4)	560 (85.9)<br>1459 (89.6)	1.4 (1.1–1.9)<br>1	0.013	1.5 (1.1–2.0)<br>1	0.006
**Parental hypertension**<br>Yes<br>No	198 (8.7)<br>2083 (91.3)	30 (15.2)<br>232 (11.1)	168 (84.8)<br>1851 (88.9)	1.4 (0.9–2.2)<br>1	0.091	–	–
**Parental diabetes mellitus**<br>Yes<br>No	70 (3.1)<br>2211 (96.9)	5 (7.1)<br>257 (11.6)	65 (92.9)<br>1954 (88.4)	1<br>1.7 (0.7–4.3)	0.247	–	–
**Modes of transport to and from school**<br>Walking/Bicycle<br>Motorcycle/car/bus	542 (23.8)<br>1739 (76.2)	40 (7.4)<br>222 (12.8)	502 (92.6)<br>1517 (87.2)	1<br>1.8 (1.3–2.6)	0.001	1<br>1.5 (1.1–2.2)	0.023
**Doing exercise/playing outdoor game**<br>Always/mostly<br>Sometimes/rarely	724 (31.7)<br>1557 (68.3)	97 (13.4)<br>165 (10.6)	627 (86.6)<br>1392 (89.4)	0.8 (0.6–1.0)<br>1	0.051	–	–
**Time spent daily on watching television**<br>< 2 hours<br>≥ 2 hours	419 (18.4)<br>1862 (81.6)	32 (7.6)<br>230 (12.4)	387 (92.4)<br>1632 (87.6)	1<br>1.7 (1.2–2.5)	0.006	1<br>1.5 (1.0–2.2)	0.063
**Consumption of snacks in between meals**<br>Always/mostly<br>Sometimes/rarely	1318 (57.8)<br>963 (42.2)	152 (11.5)<br>110 (11.4)	1166 (88.5)<br>853 (88.6)	1.0 (0.8–1.3)<br>1	0.935	–	–
**Eating confectionery/sweet foods**<br>Always/mostly<br>Sometimes/rarely	575 (25.2)<br>1706 (74.8)	60 (10.4)<br>202 (11.8)	515 (89.6)<br>1504 (88.2)	1<br>1.2 (0.9–1.6)	0.361	–	–
**Drinking soft drinks**<br>Always/mostly<br>Sometimes/rarely	1481 (64.9)<br>800 (35.1)	149 (10.1)<br>113 (14.1)	1332 (89.9)<br>687 (85.9)	1<br>0.7 (0.5–0.9)	0.004	–	–
**Frequency of consuming vegetables**<br>Always/mostly<br>Sometimes/rarely	1273 (55.8)<br>1008 (44.2)	162 (12.7)<br>100 (9.9)	1111 (87.3)<br>908 (90.1)	0.8 (0.6–1.0)<br>1	0.037	–	–

**Frequency of consuming fruit**							
Always/mostly	362 (15.9)	41 (11.3)	321 (88.7)	1	0.917	–	–
Sometimes/rarely	1919 (84.1)	221 (11.5)	1698 (88.5)	1.0 (0.7–1.5)			

AOR, Adjusted Odds Ratio; CI, confidence interval; COR, crude odds ratio; N, number.
*Variables with non-significance in Chi-square analysis were not included in logistic regression model.

Discussion

In this study, the prevalence of overweight and obesity among 2281 recruited school children of Kandahar city was 11.5%. Compared to this study, increased prevalence of overweight and obesity was present in Brazil (prevalence was 32.4% among 1125 school children aged 5.6–18 years) [3], China (prevalence was 28.2% among 3140 school children aged 7–18 years) [28], and Nepal (prevalence was 25.7% among 575 school children aged 6–13 years) [2]. Similarly, increased prevalence was reported from Tunisia (prevalence was 23.3% among 2872 boys aged 15–19 years) [29], Tanzania (prevalence was 22.6% among 1718 urban primary school children aged 8–13 years) [30], and Uganda (prevalence was 20% among 422 urban primary school children aged 7–17 years) [31]. However, a study conducted in Nigeria reported that the prevalence of overweight and obesity was 4.9% among 1187 semi-urban primary school children aged 6–11 years [8]. The inconsistence in the prevalence of overweight and obesity among these studies may be explained by differences in the study populations. It is stated that the demography of overweight/obesity prevalence differs with culture, structure, and ecology of the environment of the study populations [30].

In this study, being boy was a statistically significant risk factor for overweight/obesity in school children. Similar results have been reported in studies conducted in Iran (among 1000 urban school children aged 7–12 years) [32], China (among 3140 school children aged 7–18 years) [28], Nepal (among 575 school children aged 6–13 years) [2], and Australia (among 5252 school students aged 12–18 years) [33]. However, being girl has been reported as risk factor of overweight/obesity in studies from Tanzania (among 1718 urban primary school children aged 8–13 years) (more among girls) [30], Nigeria (among

1187 semi-urban primary school children aged 6–11 years) [8], Ghana (among 270 primary school children aged 5–15 years) [34], and Congo (among 486 adolescents) [35]. In many societies, this could be due to the restrictions on the physical activity of girls by parents [36][37]. Also, puberty causes sexual dimorphism due to which boys get more muscle-bone mass while girls gain more fat mass [38]. Among girls, early puberty is usually associated with shorter height, higher BMI, and higher risk of obesity in adulthood [38].

I this study, being student from a rich family was a risk factor for overweight/obesity. This is in accordance with the results of studies conducted in Nigeria (among 1187 semi-urban primary school children aged 6–11 years) [8], Ghana (among 270 primary school children aged 5–15 years) [34], Tunisia (among 1295 boys aged 15–19 years) [29], and Brazil (prevalence was 32.4% among 1125 school children aged 5.6–18 years) [3]. Contrary to this, a study in the United States revealed that prevalence of overweight decreased with increasing income among 7135 non-Hispanic white Americans aged 12–20 years [39]. Also a study in Norway among 15966 school students aged 15–16 years showed that poor family economy was significantly associated with overweight and obesity [40]. The above-mentioned differences in the prevalence patterns could be due to differences in the lifestyle between developed and developing countries [41]. In developing countries, as compared to poor families, rich families have better access to meat and other energy-dense fast foods, which are more expensive than other foods like vegetables [41]. Fast foods are more popular due to easy accessibility, sweet and better taste, occupation of the parents, increased socio-economic status of the family, and effects of social media [42]. Contrary to this, in developed countries, upper socio-economic class families have increased awareness of the burden of obesity and its association with unhealthy diets, usually consume more fruits and vegetables than lower socio-economic class families [41]. Also, children from rich families mostly use car for transportation, do less or no housework, and do less active work, which in turn is associated with higher risk for developing childhood obesity [28].

In this study, statistically significant increased overweight/obesity was observed in students of private schools. Similar results have been reported in studies conducted in Uganda (among 422 urban primary

school children aged 7–17 years) [31], Tanzania (among 1718 urban primary school children aged 8–13 years) (more among private students) [30], Nigeria (among 1187 semi-urban primary school children aged 6–11 years) [8], and Iran (among 1000 urban school children aged 7–12 years) [32]. Rich families usually provide more daily pocket money to their school-going children. This enables these children to eat an extra meal of junk food in the school. Also, this might be related to the fact that rich parents usually send their children to private schools in most of the developing countries [43].

In this study, statistically significant increase of overweight and obesity was observed in school children with parental obesity. A study conducted in China among 3140 school children aged 7–18 years also reported that overweight and obesity was significantly more among school students with parental obesity [28]. Genetics play an important role in obesity. So, obesity usually tracks in families. Having obese parents increases one's risk for obesity, even if family members do not live together or shares the same patterns of exercise and food intake [44–47]. However, many studies have revealed that besides the high genetic similarity among family members, parents play a crucial role in the development of children's physical activity patterns [48], eating behavior, and attitudes [49].

In this study, statistically significant increased prevalence of overweight and obesity was present among children whose mode of transportation to and from school was bus, car or motorcycle than those children who were walking. This is in accordance with the studies conducted in Uganda (among 422 urban primary school children aged 7–17 years) [31], Nepal (among 575 school children aged 6–13 years) [2], Ghana (among 400 primary school children aged 6–12 years) [50], and China (among 3140 school children aged 7–18 years) [28]. Contrary to the results of this study, studies conducted in Ghana (among 400 school-age children) [51] and Portugal (among 1786 school children aged 6–13 years) [52] did not find any significant association between modes of transportation to school and overweight/obesity among primary school children. Health promotion interventions should address childhood overweight and obesity to promote a culture of walking as a good practice among primary school children. Both parents and children should be sensitized about the health benefits of walking compared to bus, car, or

motorcycle. Also, roads should have pedestrian lanes, which will enable children to safely walk to and from school [31].

Strengths and Limitations

First and main strength of this study is that it is the first study of its type not only form Kandahar city but from the entire Afghanistan to investigate the prevalence and risk factors of overweight and obesity among school children. Second, data was randomly collected from ten (five government and five private) schools helped us in generalizing the results of this study to the school students of entire Kandahar city.

There were several limitations of this study. First, that this study was conducted in school children of Kandahar city. So, its findings may not reflect the overall scenario of the entire country and may be not generalized to the school children in rural areas. Second, information about variables related to time were answered from memory. So, recall bias cannot be ruled out. Third, as this was a cross-sectional, a causal link between the variables could not be determined. Nevertheless, this study reported probable risk factors of overweight and obesity in school children which can further be studied in the future by longitudinal studies.

Conclusion and recommendations

Prevalence of overweight and obesity was 11.5% among school children of Kandahar city. Main factors associated with overweight and obesity in school children were being boy, from rich family, student of private school, and having parental obesity. In Afghanistan, the currently focused issue is only undernutrition. However, based on the results of this study, overweight/obesity should be dealt with as an emerging problem in school children of Kandahar city.

It is recommended that Afghanistan ministries of education and public health, with the help of international donor agencies, such as WHO and UNICEF, work together in controlling overweight and obesity alongside the currently-focused undernutrition in school children of Kandahar city. Periodic special awareness programs on the prevention and control of overweight/obesity should be conducted in

schools, radio, television, and other sources of social media. Further studies are needed to be conducted in school children of rural areas in Kandahar province. Also, conduction of nationwide multicenter longitudinal studies in all 34 provinces of Afghanistan are crucial to see the prevalence of overweight/obesity not only among school children, but also in adults and elderly populations.

Abbreviations

**Abbreviation**	**Full form**
AOR	Adjusted odds ratio
BAZ	BMI-for-age Z score
BMI	Body mass index
CI	Confidence interval
COR	Crude odds ratio
HAZ	height-for-age Z score
SD	Standard deviation
SPSS	Statistical Package for the Social Sciences
UNICEF	United Nations Children's Fund
USD	United States Dollar
WFP	World Food Program
WHO	World Health Organization
WAZ	Weight-for-age Z score
WHZ	Weight-for-height Z score

Acknowledgements

We are sincerely thankful of the authorities of Kandahar University, Kandahar Directorate of Education, and the enrolled schools. We are also thankful of all the data collectors and our study participants along with their parents.

Authors' contributions

Conceptualisation: BAR, AAK, WAK, JAR, WRT.

Data curation: BAR, AAK, WAK, JAR.

Formal analysis: BAR, AAK.

Funding acquisition: No funding

Investigation: BAR, AAK, WAK, JAR.

Methodology: BAR.

Project administration: BAR, AAK.

Resources: BAR, AAK, WAK, JAR.

Software: BAR, AAK.

Supervision: BAR, AAK.

Validation: BAR.

Visualisation: BAR, WAK.

Writing – original draft: BAR.

Writing – review & editing: BAR, AAK, WAK, JAR, WRT.

All authors have read and approved the manuscript.

Funding

This study did not receive any specific funding. W. R. Taylor is part-funded by Wellcome under grant 220211. For the purposes of Open Access, the author has applied a CC BY public copyright licence to any Author Accepted Manuscript version arising from this submission.

Competing interests

All the authors do not have any competing interests.

Patient and public involvement

It was not appropriate or possible to involve patients or the public in the design, or conduct, or reporting, or dissemination plans of our research.

Consent to participate

Prior to the data collection, written informed consent from parents, and written informed assent from study participants were obtained with the help of school authorities. Interviews of participants using a predesigned questionnaire were conducted and anthropometric measurements were done as per standard operating procedures. For data collection, only patients' initials were used. Information of the participants would not be disclosed. Prior to entering into the computer for analysis, the collected data was coded and de-identified.

Ethical Approval

Ethical clearance was obtained from Ethics Committee of Kandahar University (code number KDRU-EC-2023.17) and permission from Kandahar Province Education Department authorities.

Data availability statement

All the data and materials related to this study are available on request to the corresponding author.

References

1. Ng M, Fleming T, Robinson M, Thomson B, Graetz N, Margono C, et al. Global, regional, and national prevalence of overweight and obesity in children and adults during 1980-2013:

a systematic analysis for the Global Burden of Disease Study 2013. Lancet. 2014;384: 766–781. doi:10.1016/S0140-6736(14)60460-8

2. Karki A, Shrestha A, Subedi N. Prevalence and associated factors of childhood overweight/obesity among primary school children in urban Nepal. BMC Public Health. 2019;19: 1055. doi:10.1186/s12889-019-7406-9

3. Silva AP da, Feilbelmann TCM, Silva DC, Palhares HMC, Scatena LM, Resende EAMR de, et al. Prevalence of overweight and obesity and associated factors in school children and adolescents in a medium-sized Brazilian city. Clinics (Sao Paulo). 2018;73: e438. doi:10.6061/clinics/2018/e438

4. NCD Risk Factor Collaboration (NCD-RisC). Worldwide trends in body-mass index, underweight, overweight, and obesity from 1975 to 2016: a pooled analysis of 2416 population-based measurement studies in 128·9 million children, adolescents, and adults. Lancet. 2017;390: 2627–2642. doi:10.1016/S0140-6736(17)32129-3

5. Yusuf ZI, Dongarwar D, Yusuf RA, Bell M, Harris T, Salihu HM. Social Determinants of Overweight and Obesity Among Children in the United States. Int J MCH AIDS. 2020;9: 22–33. doi:10.21106/ijma.337

6. Paduano S, Borsari L, Salvia C, Arletti S, Tripodi A, Pinca J, et al. Risk Factors for Overweight and Obesity in Children Attending the First Year of Primary Schools in Modena, Italy. J Community Health. 2020;45: 301–309. doi:10.1007/s10900-019-00741-7

7. Ramachandran A, Snehalatha C, Vinitha R, Thayyil M, Kumar CKS, Sheeba L, et al. Prevalence of overweight in urban Indian adolescent school children. Diabetes Res Clin Pract. 2002;57: 185–190. doi:10.1016/s0168-8227(02)00056-6

8. Adetunji AE, Adeniran KA, Olomu SC, Odike AI, Ewah-Odiase RO, Omoike IU, et al. Socio-demographic factors associated with overweight and obesity among primary school children in semi-urban areas of mid-western Nigeria. PLoS One. 2019;14: e0214570. doi:10.1371/journal.pone.0214570

9. Musa DI, Toriola AL, Monyeki MA, Lawal B. Prevalence of childhood and adolescent overweight and obesity in Benue State, Nigeria. Trop Med Int Health. 2012;17: 1369–1375. doi:10.1111/j.1365-3156.2012.03083.x

10. Jafar TH, Qadri Z, Islam M, Hatcher J, Bhutta ZA, Chaturvedi N. Rise in childhood obesity with persistently high rates of undernutrition among urban school-aged Indo-Asian children. Arch Dis Child. 2008;93: 373–378. doi:10.1136/adc.2007.125641

11. Kapoor SK, Anand K. Nutritional transition: a public health challenge in developing countries. J Epidemiol Community Health. 2002;56: 804–805. doi:10.1136/jech.56.11.804

12. Bray GA. Medical Consequences of Obesity. The Journal of Clinical Endocrinology & Metabolism. 2004;89: 2583–2589. doi:10.1210/jc.2004-0535

13. Sidik SM, Ahmad R. Childhood obesity: contributing factors, consequences and intervention. Malays J Nutr. 2004;10: 13–22.

14. Chen J-L, Weiss S, Heyman MB, Lustig R. Risk factors for obesity and high blood pressure in Chinese American children: maternal acculturation and children's food choices. J Immigr Minor Health. 2011;13: 268–275. doi:10.1007/s10903-009-9288-x

15. Crispim PAA, Peixoto M do RG, Jardim PCBV. Risk Factors Associated with High Blood Pressure in Two-to Five-Year-Old Children. Arquivos brasileiros de cardiologia. 2014;102: 39. doi:10.5935/abc.20130227

16. Ogden CL, Carroll MD, Curtin LR, McDowell MA, Tabak CJ, Flegal KM. Prevalence of overweight and obesity in the United States, 1999-2004. JAMA. 2006;295: 1549–1555. doi:10.1001/jama.295.13.1549

17. Dehghan M, Akhtar-Danesh N, Merchant AT. Childhood obesity, prevalence and prevention. Nutr J. 2005;4: 24. doi:10.1186/1475-2891-4-24

18. Rahimi BA, Khalid AA, Lali WM, Khalid WA, Rahimi JA, Taylor WR. Prevalence and associated risk factors of stunting, wasting/thinness, and underweight among primary school children in Kandahar City, Afghanistan: a cross-sectional analytical study. BMC Public Health. 2024;24: 2321. doi:10.1186/s12889-024-19858-z

19. Rahimi BA, Rafiqi N, Tareen Z, Kakar KA, Wafa MH, Stanikzai MH, et al. Prevalence of soil-transmitted helminths and associated risk factors among primary school children in Kandahar, Afghanistan: A cross-sectional analytical study. PLoS Negl Trop Dis. 2023;17: e0011614. doi:10.1371/journal.pntd.0011614

20. Rahimi BA, Niazi N, Rahimi AF, Faizee MI, Khan MS, Taylor WR. Treatment outcomes and risk factors of death in childhood tuberculous meningitis in Kandahar, Afghanistan: a

prospective observational cohort study. Transactions of the Royal Society of Tropical Medicine and Hygiene. 2022;116: 1181–1190. doi:10.1093/TRSTMH/TRAC066

21. Rahimi BA, Mahboobi BA, Wafa MH, Sahrai MS, Stanikzai MH, Taylor WR. Prevalence and associated risk factors of soil-transmitted helminth infections in Kandahar, Afghanistan. BMC infectious diseases. 2022;22: 361. doi:10.1186/S12879-022-07336-Z

22. Rahimi BA, Ishaq N, Mudaser GM, Taylor WR. Outcome of acute bacterial meningitis among children in Kandahar, Afghanistan: A prospective observational cohort study. Adam RD, editor. PloS one. 2022;17: e0265487. doi:10.1371/JOURNAL.PONE.0265487

23. Rahimi BA, Mohammadi E, Stanikzai MH, Wasiq AW. Determinants of Exclusive Breastfeeding Practices in Kandahar, Afghanistan: A Cross-Sectional Analytical Study. International Journal of Pediatrics. 2020;8: 11125–11140. doi:10.22038/IJP.2019.42987.3601

24. Estecha Querol S, Iqbal R, Kudrna L, Al-Khudairy L, Gill P. The Double Burden of Malnutrition and Associated Factors among South Asian Adolescents: Findings from the Global School-Based Student Health Survey. Nutrients. 2021;13: 2867. doi:10.3390/nu13082867

25. Afghanistan Ministry of Public Health and UNICEF. Afghanistan National Nutrition Survey (NNS) 2013. 2014 [cited 8 Aug 2024]. Available: https://reliefweb.int/report/afghanistan/national-nutrition-survey-afghanistan-2013

26. The World Bank. Fact Sheet: An Adjustment to Global Poverty Lines. In: World Bank [Internet]. 21 Mar 2022 [cited 24 Feb 2024]. Available:

https://www.worldbank.org/en/news/factsheet/2022/05/02/fact-sheet-an-adjustment-to-global-poverty-lines

27. de Onis M, Onyango AW, Borghi E, Siyam A, Nishida C, Siekmann J. Development of a WHO growth reference for school-aged children and adolescents. Bull World Health Organ. 2007;85: 660–667. doi:10.2471/BLT.07.043497

28. Andegiorgish AK, Wang J, Zhang X, Liu X, Zhu H. Prevalence of overweight, obesity, and associated risk factors among school children and adolescents in Tianjin, China. Eur J Pediatr. 2012;171: 697–703. doi:10.1007/s00431-011-1636-x

29. Aounallah-Skhiri H, Romdhane HB, Traissac P, Eymard-Duvernay S, Delpeuch F, Achour N, et al. Nutritional status of Tunisian adolescents: associated gender, environmental and socio-economic factors. Public Health Nutrition. 2008;11: 1306–1317. doi:10.1017/S1368980008002693

30. Pangani IN, Kiplamai FK, Kamau JW, Onywera VO. Prevalence of Overweight and Obesity among Primary School Children Aged 8–13 Years in Dar es Salaam City, Tanzania. Adv Prev Med. 2016;2016: 1345017. doi:10.1155/2016/1345017

31. Twinomugisha B, Birungi AM. Prevalence and Associated Factors of Overweight and Obesity among Primary School Children Aged 7–17 Years in Urban Mbarara, Uganda. Adv Public Health. 2024;2024: 5175550. doi:10.1155/2024/5175550

32. Hajian-Tilaki KO, Sajjadi P, Razavi A. Prevalence of overweight and obesity and associated risk factors in urban primary-school children in Babol, Islamic Republic of Iran. East Mediterr Health J. 2011;17: 109–114.

33. O'Dea JA. Gender, ethnicity, culture and social class influences on childhood obesity among Australian schoolchildren: implications for treatment, prevention and community education. Health Soc Care Community. 2008;16: 282–290. doi:10.1111/j.1365-2524.2008.00768.x

34. Mohammed H, Vuvor F. Prevalence of childhood overweight/obesity in basic school in Accra. Ghana Med J. 2012;46: 124–127.

35. Longo-Mbenza B, Lukoki Luila E, M'Buyamba-Kabangu JR. Nutritional status, socio-economic status, heart rate, and blood pressure in African school children and adolescents. Int J Cardiol. 2007;121: 171–177. doi:10.1016/j.ijcard.2006.11.004

36. Sweeting HN. Gendered dimensions of obesity in childhood and adolescence. Nutr J. 2008;7: 1. doi:10.1186/1475-2891-7-1

37. Goran MI. Metabolic precursors and effects of obesity in children: a decade of progress, 1990-1999. Am J Clin Nutr. 2001;73: 158–171. doi:10.1093/ajcn/73.2.158

38. Stein AD, Lundeen EA, Martorell R, Suchdev PS, Mehta NK, Richter LM, et al. Pubertal Development and Prepubertal Height and Weight Jointly Predict Young Adult Height and Body Mass Index in a Prospective Study in South Africa. J Nutr. 2016;146: 1394–1401. doi:10.3945/jn.116.231076

39. Gordon-Larsen P, Adair LS, Popkin BM. The relationship of ethnicity, socioeconomic factors, and overweight in US adolescents. Obes Res. 2003;11: 121–129. doi:10.1038/oby.2003.20

40. Grøholt E-K, Stigum H, Nordhagen R. Overweight and obesity among adolescents in Norway: cultural and socio-economic differences. J Public Health (Oxf). 2008;30: 258–265. doi:10.1093/pubmed/fdn037

41. Wang Y. Cross-national comparison of childhood obesity: the epidemic and the relationship between obesity and socioeconomic status. Int J Epidemiol. 2001;30: 1129–1136. doi:10.1093/ije/30.5.1129

42. Das J. Fast Food Consumption in Children: A Review. Med Clin Rev. 2015;1: 1–4. doi:10.21767/2471-299X.1000001

43. Abah S. O, Aigbiremolen A. O, Duru Cb, Awunor Ns, Asogun Ad, Enahoro Fo, et al. Prevalence of overweight and obesity among students in private and public secondary schools in a peri-urban Nigerian town. Journal of Biology, Agriculture and Healthcare. 2012;2: 51.

44. Bogaert N, Steinbeck KS, Baur LA, Brock K, Bermingham MA. Food, activity and family--environmental vs biochemical predictors of weight gain in children. Eur J Clin Nutr. 2003;57: 1242–1249. doi:10.1038/sj.ejcn.1601677

45. Li Y, Zhai F, Yang X, Schouten EG, Hu X, He Y, et al. Determinants of childhood overweight and obesity in China. Br J Nutr. 2007;97: 210–215. doi:10.1017/S0007114507280559

46. Urakami T, Morimoto S, Nitadori Y, Harada K, Owada M, Kitagawa T. Urine glucose screening program at schools in Japan to detect children with diabetes and its outcome-

incidence and clinical characteristics of childhood type 2 diabetes in Japan. Pediatr Res. 2007;61: 141–145. doi:10.1203/pdr.0b013e31802d8a69

47. Wu Y, Huxley R, Li M, Ma J. The growing burden of overweight and obesity in contemporary China. CVD Prevention and Control. 2009;4: 19–26. doi:10.1016/j.cvdpc.2008.11.003

48. Lyon HN, Hirschhorn JN. Genetics of common forms of obesity: a brief overview. Am J Clin Nutr. 2005;82: 215S-217S. doi:10.1093/ajcn/82.1.215S

49. Farooqi IS, O'Rahilly S. Genetic factors in human obesity. Obes Rev. 2007;8: 37–40. doi:10.1111/j.1467-789X.2007.00315.x

50. Amidu N, Owiredu W, Saaka M, Quaye L, Wanwan M, Kumibea PD, et al. Determinants of childhood obesity among basic school children aged 6 – 12 years in Tamale Metropolis. Journal of Medical and Biomedical Sciences. 2013;2: 26–34. doi:10.4314/jmbs.v2i3.5

51. Mogre V. Physical Activity and BMI Status of School-Age Children in Tamale, Northern Ghana. Pak J Nutr. 2013;12: 484–490.

52. Vasques C, Mota M, Correia T, Lopes V. Prevalence of overweight/obesity and its association with sedentary behavior in children. Rev Port Cardiol. 2012;31: 783–788. doi:10.1016/j.repc.2012.03.005
